# Public mental health through social media in the post COVID-19 era

**DOI:** 10.3389/fpubh.2023.1323922

**Published:** 2023-12-11

**Authors:** Deepika Sharma, Jaiteg Singh, Babar Shah, Farman Ali, Ahmad Ali AlZubi, Mallak Ahmad AlZubi

**Affiliations:** ^1^Chitkara University Institute of Engineering and Technology, Chitkara University, Punjab, India; ^2^College of Technological Innovation, Zayed University, Dubai, United Arab Emirates; ^3^Department of Computer Science and Engineering, School of Convergence, College of Computing and Informatics, Sungkyunkwan University, Seoul, Republic of Korea; ^4^Department of Computer Science, Community College, King Saud University, Riyadh, Saudi Arabia; ^5^Faculty of Medicine, Jordan University of Science and Technology, Irbid, Jordan

**Keywords:** public mental health, individual behavior, micro-expressions, COVID-19, social media, CNN

## Abstract

Social media is a powerful communication tool and a reflection of our digital environment. Social media acted as an augmenter and influencer during and after COVID-19. Many of the people sharing social media posts were not actually aware of their mental health status. This situation warrants to automate the detection of mental disorders. This paper presents a methodology for the detection of mental disorders using micro facial expressions. Micro-expressions are momentary, involuntary facial expressions that can be indicative of deeper feelings and mental states. Nevertheless, manually detecting and interpreting micro-expressions can be rather challenging. A deep learning HybridMicroNet model, based on convolution neural networks, is proposed for emotion recognition from micro-expressions. Further, a case study for the detection of mental health has been undertaken. The findings demonstrated that the proposed model achieved a high accuracy when attempting to diagnose mental health disorders based on micro-expressions. The attained accuracy on the CASME dataset was 99.08%, whereas the accuracy that was achieved on SAMM dataset was 97.62%. Based on these findings, deep learning may prove to be an effective method for diagnosing mental health conditions by analyzing micro-expressions.

## 1 Introduction

The interconnectedness of individual behavior, the environment, COVID-19, and social media has significantly shaped the global community during the COVID-19 pandemic. As a powerful communication tool and a reflection of our digital environment, social media acted as an augmenter and influencer during and after COVID-19. Social media platforms have become primary sources of information about COVID-19. Social media platforms primarily share news, updates, and expert opinions. The rapid spread of information on social media has resulted in the promulgation of misinformation, conspiracy theories, and false claims about COVID-19.

Social media served as a platform for sharing images, videos, and stories related to the pandemic. These visuals spread awareness about the environmental impact of the virus, including healthcare systems working beyond their capacities and shortages of medical supplies. Social media was instrumental in organizing community efforts during the pandemic, ranging from neighborhood support groups to initiatives for assisting vulnerable populations (1). Social media provides a direct channel for government agencies and public health organizations to communicate with the public and offers spaces for sharing personal experiences and seeking emotional support. The pandemic has taken a toll on mental health, and social media has played a role in exacerbating stress and offering a platform for coping mechanisms. Social media platforms have also been responsible for creating echo chambers where individuals are exposed only to information that aligns with their preexisting beliefs. Excessive use of social media can contribute to mental health issues, such as anxiety, depression, and social isolation (2, 3). The constant streaming of pandemic-related news on social media can lead to information overload and increased stress. It may influence individual behavior and mental health. Understanding the interconnectedness of individual behavior, the environment, COVID-19, and social media is critical for policymakers, public health officials, and researchers. It underscores the importance of managing information dissemination, addressing misinformation, and leveraging social media to promote positive behaviors and community resilience. Moreover, it highlights the need for a holistic approach to public health that considers the digital environment an integral part of the broader ecosystem in which health crises unfold (4, 5).

The transformations occurring within the broader community have played a substantial role in escalating mental health issues and behavioral disorders. The World Health Organization (WHO) has defined “mental health” as the state of an individual who can effectively manage their life stressors within their capabilities while simultaneously maintaining optimal functioning in their occupational pursuits and making meaningful contributions to society (6). Various elements that impact mental health likely emanate from an individual’s lifestyle, encompassing work-related stress, unfavorable financial circumstances, familial challenges, interpersonal difficulties, and exposure to violence. Additionally, environmental factors may also contribute to these effects (7). Mental health is becoming an enormous global issue, considering the millions of individuals affected by mental illnesses, including stress, anxiety, Post-Traumatic Stress Disorder (PTSD), and depression (8). Many people remain unidentified or unattended, even though early identification and assistance are essential for the optimal treatment and supervision of such disorders. Conventional techniques for identifying mental health issues, such as clinical investigations, self-report questionnaires, psychological evaluations, and professional interviews, can be biased, costly, and time-consuming. These methods might not be accurate or dependable, particularly for those unwilling to express their genuine emotions (9). Since emotions are a fundamental part of human interaction, they can significantly impact mental illnesses in the context of human behavior, responses, and perspectives. Anger, disgust, sadness, and fear are some negative emotions that people with mental conditions may encounter. Such emotions can be intense and persistent, making it challenging for the individual experiencing them to carry on with their regular activities (10). Over the conventional techniques, however, there has been a dearth of appropriately trained mental health specialists, and the stigma prevents many people from seeking assistance. However, concerning the traditional methods for diagnosing mental disorders, there is a shortage of adequately trained mental health specialists, and the stigma keeps many people from getting help (11).

Alternatively, new approaches to the large-scale identification and understanding of emotions and mental health conditions have been made possible by recent developments in computer vision, affective computing, human-computer interaction, machine learning, and information analytics techniques. Machine learning techniques are fully dependent on training and testing data, which can be collected through Internet of Things (IoT) devices, social media networking sites, physiological signals, facial expressions, speech, and text (12–14). Social networking platforms such as YouTube, Twitter, Facebook, Sina Weibo, Instagram, and Reddit that allow users to express their thoughts and emotions are becoming more and more popular. Social media platforms allow users to express their emotions and thoughts by sharing various forms of data, including text, photographs, audio, and videos, pertaining to their everyday experiences. Millions of individuals worldwide daily utilize these social networking platforms. Various forms of data, including text, photos, video, and audio, convey feelings and thoughts through online postings. These applications generate a substantial amount of data. This overabundance of data results from a growth in communication, and these massive amounts of data have become content producers that could be helpful for additional research and analysis. As a result, it can be utilized to create a method for identifying mental health issues. The analysis of facial expressions and their correlation with personal conceptions is a pertinent issue in social media, as facial expressions have been significant indicators of various psychological characteristics in human beings (15).

Facial micro-expression analysis has gained popularity in identifying mental health issues in recent years. Microexpressions are quick, uncontrollable facial gestures that convey a person’s subconscious feelings. They seem to be a potentially helpful tool for mental health detection because they are hard to manipulate or fabricate (16, 17).

The researchers have created machine-learning models that can identify microexpressions linked to sadness, anxiety, and depression with a high degree of precision. These models can be applied to developing novel diagnostic frameworks for the early identification and treatment of mental health issues. Additionally, new diagnostic instruments for mental health practitioners could be developed by employing deep learning models. For instance, a model might be used to interpret a patient’s facial expressions during a clinical interview to assist the physician in diagnosing the patient (15, 17–19). Microexpressions are unintentionally reflected expressions that cannot be seen through the naked eye as compared to macroexpressions. Micro-expressions can be captured through a high-resolution camera (20). The proposed study presents a framework for analyzing the facial micro-expressions in video frames of people with mental disorders and healthy controls by analyzing their social media videos.

The structure of this document is as follows: Section 2 includes related work, Section 3 contains information on the collection and pre-processing of the data and proposed Hy-bridMicroNet model used for emotion recognition, Section 4 presents the experimental work along with results and discussion, Section 5 embodies the case study and Section 6 provides the conclusion and future scope.

## 2 Literature review

The integration of micro-expressions for the identification of mental health illnesses has become an important subject in computer science and psychology. A summary of significant studies and developments in the field of micro-expressions based mental health detection is given in this related work section. Paul Ekman’s research on micro-expressions, which reveal concealed emotions and emotional disorders, has paved the way for the potential use of these expressions for mental health assessment (21).

Researchers investigated into using micro-expressions as a technique to detect depression, which is among the most common mental health disorders. Facial dynamics, vocal parodies and head motions were employed in clinical interviews and interpersonal settings to determine the level of depression that was identified in the research study. In all, 57 patients with depression were asked to take part in the investigation, and the Hamilton depression rating scale was used to conduct their assessments. Using Z-Face technology, 3D registration from the 2D video is accomplished in this work. Stack Denoising Auto-encoders (SDAE) are then employed to encode the head and face movements by mapping features and enhancing fisher vector coding. This research has a 78.67% accuracy rate (22).

Beck Depression Inventory (BDI) score estimation used for feature analysis of facial expressions, employed for automatic depression detection. Macro-structure and Micro-structure of facial dynamics features were extracted through Median Robust Local Binary Patterns from three orthogonal Planes (MRLBP-OP). The MRLBP-Top across an image succession was also suggested to be aggregated using Dirichlet Process Fisher Encoding (DPFP). The assessments and evaluations were conducted using the depression datasets AVEC-2013 and AVEC-2014. According to the Mean Absolute Error (MAE) of 7.55 and the Root Mean Square Error (RMSE) of 9.20, the findings were assessed (23).

In a subsequent study, BDI was used to detect depression levels employing the video data. Recurrent neural networks and 3D convolution neural networks (RNNsC3D) were applied in the proposed framework for automatically learning the spatiotemporal characteristics of the facial region. AVEC-2013 and AVEC-2014 depression datasets were used for the experiments. The findings that were obtained have been assessed using the RMSE with 9.28 and MAE with 7.37 (24).

A deeper and naive model was proposed to classify and quantify depression based on textual and visual descriptions. Text and audio-visual features were extracted using Support Vector Machine (SVM), and a deep Convolutional Neural Network (CNN) for evaluation of physical and mental health of the individual. Random Forest (RF) was applied to classify the depression utilizing the inputs from SVM and CNN. The research findings obtained using AVEC-2016 dataset, having a the F1 score of 0.746 (25).

Multimodal Attention Feature Fusion (MAFF) and Spatiotemporal Attention Network (STA) techniques-based model was proposed for depression detection and prediction. Audio analysis was performed by dividing the voice frequency into fixed length segments and subsequently inserting these segments to a STA network to integrate temporal and spatial data. This framework applied the eigen evolution pooling technique to evaluate the alterations in every dimension associated with the audio-visual segmentation feature. Additionally, Support Vector Regression (SVR) was employed for processing the MAFF. The experimentation yielded an RMSE of 8.16 and an MAE of 6.14 using the AVEC2013 and AVEC2014 datasets (26).

Automatically detection of severity signs of anxiety and depression were measured by proposed SVM model through features extraction techniques such as Local Binary Patterns (LBP), Histogram of Oriented Gradients (HOG), and Visual Geometry Group (VGG) from a video dataset. BDI-II and State–Trait Anxiety Inventory (STAI) scores were calculated in terms of normalized RSME (16.73, 12.42) and MAE (12.48, 9.85). The suggested approach was assessed using LeaveOne-Subject-Out (LOSO) cross-validation technique. This procedure can be repeated as per number of subjects in the dataset rather than sample collection. Gender-dependent and gender-independent cross-validation were executed out independently. The framework was trained and tested using attributes generated from either female or male subjects in the gender-dependent strategy. Findings shows that VGG-19 feature extraction technique was outperformed to detect depression and HOG also performed better in another setups. The results obtained from current dataset (depressed-20 and healthy controls-45) were compared with AVEC2014 dataset (16).

An Enriched Long-term Recurrent Convolutional Network (ELRCN) was proposed for emotion recognition through facial micro-expressions. The model has two variants for spatial feature extraction and temporal dynamics characterization named Spatial Dimension Enrichment (SE) and Temporal dimension Enrichment (TE). Large data input used for SE by stacking the optical flow, optical strain, and gray-scale images. VGG-16 model used for training the model and ending fully connected layer encoded the data into fixed- length vector. VGG-Face model used for TE for transfer learning of pre-trained weights. Both variants used Long Short-Term Memory (LSTM) algorithm for temporal learning. The Chinese Academy of Sciences Micro-expression (CASME-II) and Spontaneous Micro-Facial Movement Dataset (SAMM) datasets were used for experiment to obtain F1-score, accuracy in Weighted Average Recall (WAR) and Unweighted Average Accuracy (UAR). LOSO methodology was used for training since it minimizes subject bias in the learning process (27).

A framework for detection of Autism Spectrum Disorder (ASD) based on micro-expressions was developed using computer vision techniques. Facial Action Coding System (FACS) was used for facial micro-movements analysis. FACS contains a comprehensive set of features labeled as Action Units (AUs) that depict the individual parts of face, enabling the decoding and measurement of every potential expression due to contraction of the facial underlying muscles. This study specifically focused on positive facial expressions “smile” (connected with AU12) particularly Social Smile that can be identified by extra activation of AU06. Multivariate Analysis of Variance (MANOVA) was used to test for statistically significant differentiation between ASD and Typically Developed (TD) groups. The results indicated that statistically substantial variations between the Typically Developed (TD) and ASD groups for descriptive variables such age, gender, and home video characteristics in case of social smile (28).

To identify the older adult depression an investigation was conducted based on facial micro-expressions recognition from the video dataset of 100 older adult subjects (50 – depressed, 50 – healthy controls). The video frames were processed into an optical flow image that reflects the variations in micro-expressions. Due to rapid changes with low amplitude of micro-expressions, a jump connection VGG-16 model was employed for feature fusion on the outcomes of 3–5 blocks of convolutional layers. To identify the older adult depressive people’s microexpressions, the merged results were placed into a fully-connected layer. Mean recall and F1-score were measured for evaluation of model performance on CASME, SMIC, SFEW and VAM corpus datasets. The results demonstrate that older adult depressed individuals, regardless of gender, showed a significant difference in the occurrences of negative as well as positive expressions for each level when compared to normal people. This supports the theory that older people who are depressed respond less strongly to happy emotions and more strongly to negative ones (29).

A CNN-based method was presented to identify Major Depressive Disorder (MDD) by predicting emotion from the facial expressions of 12 subjects in YouTube interview videos. The VGG16 model was used to perform facial recognition on extracted frames, which were obtained every 0.2 s. During the data pre-processing stage, multiple faces or frames with no faces were eliminated. Using Openface and VGG19, respectively, several AUs and emotional characteristics were retrieved. SVM, LR and gradient boosted decision tree algorithm were used for classification of seven emotions. A leave-one-subject-out cross-validation approach was employed to achieve an Area Under the Curve (AUC) of 0.72 for remission categorization and 0.75 for response to therapy (30).

The COVID-19 epidemic has resulted in a surge in social media utilization, as individuals increasingly depend on digital platforms to access health-related information. Based on a comprehensive analysis of existing literature, it has been determined that heightened exposure to information related to COVID-19 through various channels such as mass media and social media platforms is strongly correlated with an increasing prevalence of psychological health concerns (31). There have been worries raised over the potential influence of social media on mental health amidst the ongoing pandemic. Several studies have established a correlation between heightened use of social media platforms and an elevated susceptibility to depression. Additionally, exposure to adverse news articles and postings has been identified as a contributing factor to the risk of depression in certain individuals (32). The proliferation of misinformation on the internet, commonly referred to as the “infodemic,” encompasses the dissemination of inaccurate information pertaining to COVID-19, such as unverified remedies, the propagation of anti-Asian sentiments, and the circulation of conspiracy theories. This phenomenon has been identified as a contributing factor to heightened levels of anxiety and stress among those who are already grappling with the impacts of the pandemic (33). The present study conducted a meta-analysis of 14 cross-sectional studies, which collectively examined the relationship between excessive usage of social media platforms and the prevalence of anxiety and depressive symptoms among the general population. The findings of this meta-analysis indicate a significant positive association between the amount of time individuals spend on social media platforms and the likelihood of experiencing heightened levels of anxiety and depressive symptoms (3). Nevertheless, several research have indicated that social media can serve as an adaptive mechanism for coping, leading to a decrease in stress and anxiety levels. Social media has been recognized as a potential mechanism for augmenting social support among individuals who face challenges in finding sufficient offline social support networks (34, 35). The user’s text does not contain any information to rewrite. The scholarly literature has examined the changes in social media behavior pre- and post-SARS-CoV-2 infection, as well as explored the influence of social media on mental health in the context of the COVID-19 pandemic (34, 36, 37).

The time frame adhering to the COVID-19 pandemic has initiated a significant shift in the complex interplay between mental health and the patterns of social media. The emergence of the COVID-19 pandemic led to a significant spike of internet connectivity, resulting in a huge rise in the usage of social media platforms. A comprehensive comprehension of this intricate concept is crucial in the formulation of precise interventions that effectively utilize the advantageous aspects of social media, while concurrently minimizing the possible adverse impacts on mental well-being.

The present work provides a comparative analysis of contemporary research studies, focusing on their methodological foundations, dataset features, accuracy measures, and inherent limits. Through a comparative analysis of these fundamental elements, it is possible to ascertain the merits and limitations of each methodology, so cultivating a holistic comprehension of the existing scholarly terrain. Table 1 presents a comprehensive summary of these aspects, facilitating the comprehension of readers regarding the intricacies and consequences of each study.

**Table 1:** Comparative analysis of contemporary research studies.

Ref.	Mental disorder	Methodology	Dataset	Accuracy	Limitation
(22)	Depression	Clinical interviews, Hamilton depression rating scale, Z-Face technology, SDAE	Self	78.67%	Small sample size, no healthy control participation
(23)	Depression	BDI score, MRLBP-TOP, Support Vector Regression, GMM	AVEC-2013, AVEC-2014	MAE-7.55 RSME-9.20	Lack of consideration of gender, age and ethnicity for assessment
(24)	Depression	3D-RNN, convolutional 3D network, BDI-II Score	AVEC-2013, AVEC-2014	MAE-7.37 RSME-9.28	Facial analysis done only on loose-face and tight-face, No emotion classification
(25)	Depression	SVM, CNN, RF	AVEC-2016	F1 score-0.746	Small sample size
(26)	Depression	MAFF, STA, SVR, BDI-II score	AVEC-2013, AVEC-2014	MAE-6.14 RSME-8.16	Model accuracy can be affected by noisy & low-quality data Results may vary in real-world clinical settings
(16)	Anxiety and Depression	LBP, VGG, HOG, BDI-II, SATI, SVM	AVEC-2014	MAI-12.48, 9.48 RSME-16.73, 12.42	Small sample size, Accurcy depends upon the video quality, results may differ on different population
(27)	Emotion Recognition	VGG-16, LSTM, Recurrent CNN	CASME-II, SAMM	F1-score − 0.50 WAR-0.5244 UAR-0.4396	It contains a large number of parameters may increase the risk of overfitting
(28)	ASD	CNN, HOG, SVM, SVR, MANOVA	Self	F1-score (1, 27) for ASD & TD	Small sample size, Limited data collection, lack of longitudinal data, lack of consideration of cultural factors
(29)	older adult Depression	VGG-16,	Self, CASME, SMIC, SFEW, VAM	F1-score Mean Recall	Limited range of micro-expressions, results can be biased due to age-related changes in facial appearance
(30)	MDD	VGG-19, SVM, LR,	12-subjects videos from youtube	AUC -0.72	Small sample size, cross-cultural differences can affect the results accuracy

## 3 Proposed method

**Figure 1:**
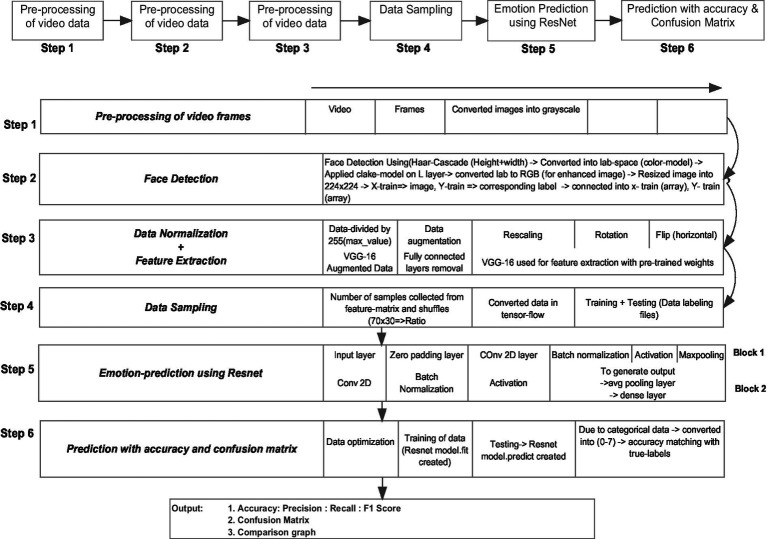
Proposed HybridMicroNet framework architecture.

The proposed HybridMicroNet architecture proposed to recognize facial microexpressions using an improved deep-learning network is shown in Figure 1. It is designed to effectively capture and analyze subtle micro-expressions, along with a hybrid novel feature extraction approach. The model consists of multiple layers that extract and process facial features at a granular level, enabling accurate emotion detection. The proposed framework architecture is divided into four phases. The first phase is related to extracting video frames (10 frames per second) from an input video. The second phase involves converting the retrieved images from the video to grayscale, applying the Haar-cascade classifier to identify faces, and then resizing the image to 224 × 224. The third phase involves extracting features using the VGG-16 model with pre-trained weights. To predict the emotion at the final phase, a hybrid model that combines VGG-16 and ResNet is implemented.

### 3.1 Data pre-processing

The first step in pre-processing the microexpression video dataset is to divide the video into frames and conversion of these frames into grayscale. The starting and ending frame is taken equally from the input video for data normalization. Ten frames per second are collected from the video and converted into grayscale images to detect the face more precisely and clearly.

### 3.2 Face detection

The primary goal of the face detection phase is to identify the faces present in the grayscale image. A machine learning technique called the Haar-cascades approach entails developing a classifier from an extensive number of images that are both positive and negative. Positive images are related to detected human faces, and negative images without any face images. Haar features are patterns of white and dark pixels in an image that are used for further processing. The unique values of each attribute, which can identify the faces of different persons in different contexts, are calculated by deducting the sum of all the pixels in the white rectangle from the total number of pixels in the black rectangle. The captured images are enhanced by a non-linear image processing technique to improve the contrast of micro-expressions. The last step of face detection is to resize the image into 224 × 224 to be used as an input to the feature extraction phase.

### 3.3 Feature extraction

A deep convolutional neural network, the VGG-16 model, is used to extract the shape, texture, edge, color, semantic, and conceptual features from the images for micro-expression detection. VGG-16 model has been pre-trained on a large image dataset, which allows it to learn the different features. This model was initially used to predict the probability of the image pixels into specific feature category classes such as texture, edge, and shape.

### 3.4 Emotion recognition

A new architecture of ResNet model is implemented to recognize emotions, which receives the features from the VGG-16 model and is further used for training and testing of data. The proposed HybridMicroNet model takes two optional parameters: the shape of the input data and the number of output classes for classification. Fundamental characteristics are extracted from the input image by the first convolutional layer. Spatial dimensions are increased by applying zero padding to ensure the convolutional layer’s output matches the dimensions of the input image data. The padded input is fed into a 2D-convolutional layer that has batch normalization and ReLU activation. The input image with size 224 × 224 is subjected to the convolutional layer with 16 filters and a 7×7 kernel size and 2 × 2 stride size with padding 2. The result of the ReLU activation layer is fed to a max pooling layer that uses a 3×3 kernel size as well as a stride of 2.

A function has been developed that repeats a two-layer module built up of successive convolutional layers, batch normalization layers, ReLU activation layers, and another convolutional layer. This function defines the building blocks for the CNN model. It requires three parameters: filters (a list of two numbers that indicates how many filters there are for each of the two convolutional layers), f (the kernel size for convolutional layers), and X (the input tensor). A number of filters can be specified in the two convolutional layers. Additionally, the function projects the shortcut connection X_shortcut in a manner that ensures the dimensions match correctly. In case the number of filters in the shortcut (X_shortcut) differs from the number of filters in the output of the convolutional layers, a 1×1 convolutional layer is applied to X_shortcut to bring the number of filters in line. After that, the outcome is subjected to batch normalization. A residual connection is formed by adding the shortcut connection and the convolutional layer output element-wise. The obtained tensor is then returned after the sum is subjected to a ReLU activation.

The function RLayers is called twice, adding to the output of the preceding layer a predetermined pattern of convolutional layers with batch normalization. The resulting feature maps are subjected to global average pooling. The class probabilities are generated using a fully connected (FC) dense layer using a softmax activation function. Table 2 shows the network configuration of the HybridMicroNet model.

**Table 2:** Network configuration of HybridMicroNet.

Layer type	Output shape	# of parameters
Conv2D (7×7, 16 filters)	(4, 4, 16)	5024
BatchNormalization	(4, 4, 16)	64
MaxPooling2D	(1, 1, 16)	0
Conv2D (3×3, 16 filters)	(1, 1, 16)	2320
BatchNormalization	(1, 1, 16)	64
Conv2D (3×3, 16 filters)	(1, 1, 16)	2320
BatchNormalization	(1, 1, 16)	64
Add	(1, 1, 16)	0
Conv2D (3×3, 32 filters)	(1, 1, 32)	4640
BatchNormalization	(1, 1, 32)	128
Conv2D (3×3, 32 filters)	(1, 1, 32)	9248
BatchNormalization	(1, 1, 32)	128
Add	(1, 1, 32)	0
GlobalAveragePooling2D	(32,)	0
Dense (Output Layer)	(2,)	66
Total	–	23702

## 4 Experiment and results

This section may be divided by subheadings. It should provide a concise and precise description of the experimental results, their interpretation, as well as the experimental conclusions that can be drawn.

### 4.1 Datasets

A benchmark dataset CASME-II obtains 249 data samples of 26 Asian volunteers with 22.03 average age producing spontaneous micro-expressions. Every picture is 640 × 480 pixels in resolution, with the face area measuring about 280 × 340 pixels. Depending on the circumstances that most appropriated the emotional state, the dataset was acquired at different intervals of time. CASME-II is a fully labeled dataset with high resolution, which offers assurance in the algorithm’s evaluation and testing purposes. The participants were depicted in five types of micro-expressions such as Disgust, happiness, Surprise, Repression and Others (38, 39).

SAMM datasets obtains 159 samples at 200 frame per second from 32 subjects with 2040 × 1,088 resolution. The facial region area is about 400 × 400 resolution. There are seven classes of emotion with other expression such as sadness, happiness, fear, disgust, surprise, anger, contempt, and others (40).

### 4.2 Implementation

The input image with dimensions of 224 × 224 was given padding with three additional pixels on all sides, and the value 1 was assigned to each of these padded pixels. The input image with dimensions of 224 × 224 was given padding with three additional pixels on all sides, and the value 1 was assigned to each of these padded pixels. The images underwent various forms of data enhancement, such as rescaling, rotation and having their orientations flipped horizontally. Additionally, VGG-16 model last three fully connected layers were removed to obtain features extracted from images. A feedforward weight activation approach was used to generate residual attention units throughout the training process. Stochastic Gradient Descent (SGD) technique was used for training with 64 batch size. Training was carried out with the use of a technique called stochastic gradient descent (SGD), and the batch size was set to 64. A rate of 0.001 has been applied for the application of weight deterioration, while a value of 0.5 was applied for momentum. Throughout the training process, these hyperparameters determine how the model’s weights are modified. Categorical Crossentropy is a loss function that is frequently used for multiclass classification. This function refers to the idea of negative log-likelihood in order to quantify the dissimilarity that exists among predicted probabilities and the actual labels. Training of data using a batch size of 32 and ten epochs, along with a validation function to assess the performance of the model.

### 4.3 Evaluation metrics

**Figure 2:**
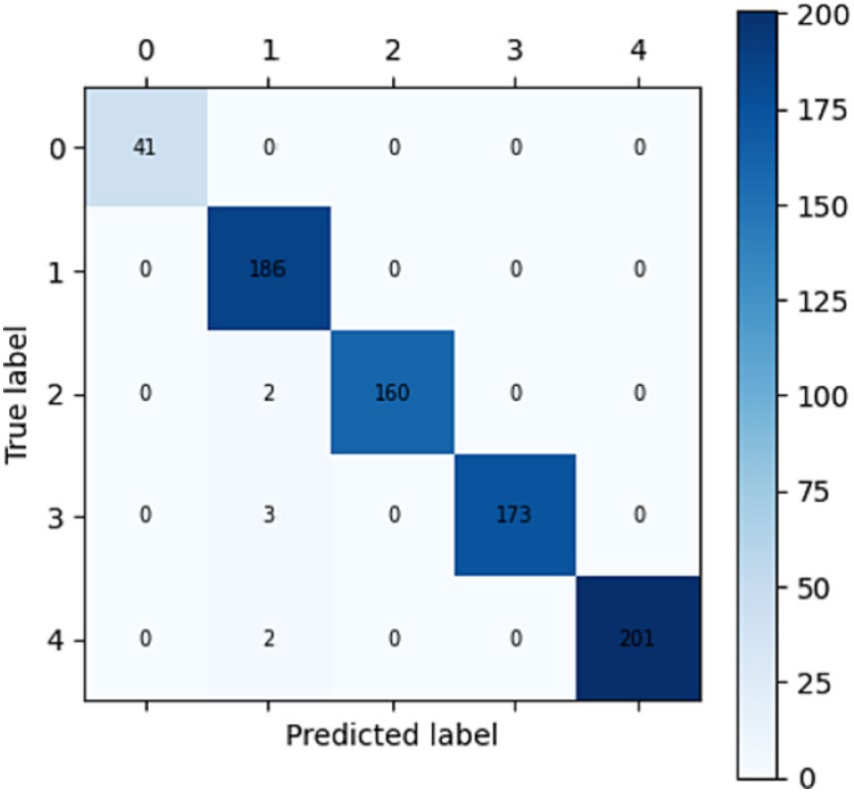
Confusion matrix for CASME-II dataset for five classes: 0-Happiness, 1-Repression, 2-Surprise, 3-Disgust, 4-others.

**Figure 3:**
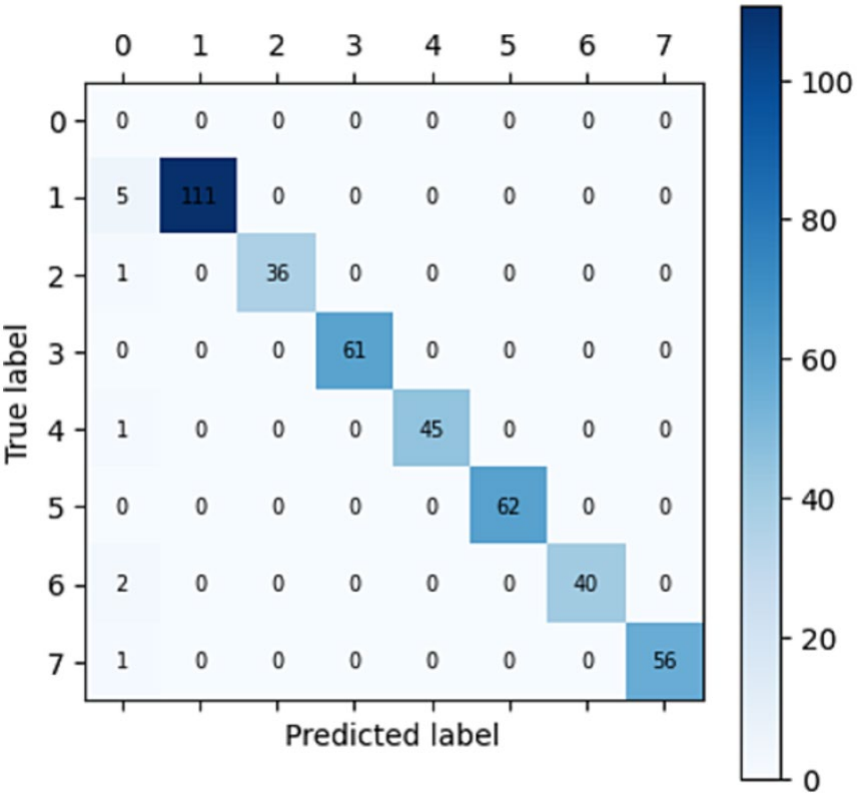
Confusion matrix for SAMM dataset with seven classes: 0-Happiness, 1-Surprise, 2-Anger, 3-Disgust, 4-Sadness, 5-Fear, 6-Contempt, and 7-Others.

To evaluate the performance of the proposed model, testing on the rest part of data (test_data) has been performed for emotion prediction. It is possible that the model is supplying probability scores or continuous values, a round function is applied to round off the nearest whole number to resolve this problem. Function assigns the test labels to all the images in which the model’s most confident prediction is for the class that has the best accuracy. This function applies the function to all the images in the dataset. CASME-II and SAMM datasets are used to predict emotions in five and seven classes through confusion metrics (shown in Figures 2, 3) and accuracy, precision, recall and F1-score (Table 3).

**Table 3:** Comparison of accuracy measures between datasets.

Dataset	Accuracy	Precision	Recall	F1-score
CASME-II	99.08	99.12	99.08	0.99
SAMM	97.62	100	97.62	0.98

### 4.4 Discussion

The comparison of proposed methodology with existing state-of-the-art approaches in the domain of machine learning-based micro-expression recognition highlights the notable progress that has been made. The advantages of proposed technique is validated through a comparative examination against established approaches, demonstrating improved precision, recall, accuracy and overall performance measures.

The LBP-TOP approach (41), involves the extraction of features into an SVM classifier. Despite outperforming hand-crafted solutions, these approaches demonstrate limitations in sustaining high levels of accuracy in real-time scenarios. In another study (42) a method introduced for deriving local quantized patterns based on spatiotemporal information. The aforementioned approach demonstrates the ability to acquire dynamic patterns; nonetheless, its performance outcomes were not deemed satisfactory. In this study (43), researcher used data augmentation methods to produce synthetic images, which were subsequently employed in the training process of a CNN. In order to accurately classify optical flow properties of facial micro expressions, it was necessary to acquire and retain crucial motion characteristics. One potential limitation of this approach is the potential loss of crucial temporal information through the generation of synthetic data.

In a further study (44), a methodology employed that encompassed the extraction of spatiotemporal data from facial images, which was subsequently processed using a three-dimensional convolutional neural network (3D CNN). The MicroExpSTCNN method encompasses the extraction of information from all pixels inside an image, whereas the MicroExpFuseNet method focuses on extracting information just from the regions of the eyes and mouth. While previous research (44) has demonstrated impressive accuracy rates when utilizing the CASME II and SMIC datasets, it is important to note that real-time prediction may still be susceptible to false positives and false negatives as a result of employing 3D CNN filters for computation. Subsequently, a three-dimensional flow approach (45) has introduced which involved employing a CNN model for the purpose of recognizing micro expressions in video-based contexts. It presents similar limitations as those discussed in reference (44). The Lateral Accretive Hybrid Network (LEARNet) (46) employs a specialized domain-specific area that incorporates depth maps and performs computations using ResNet and convolutional layers. The LEARNet image network specifically developed for the purpose of accurately identifying and analyzing micro expressions. This study presents a novel approach for capturing and preserving facial movement information in a single frame by utilizing a dynamic representation of micro-expressions.

**Figure 4:**
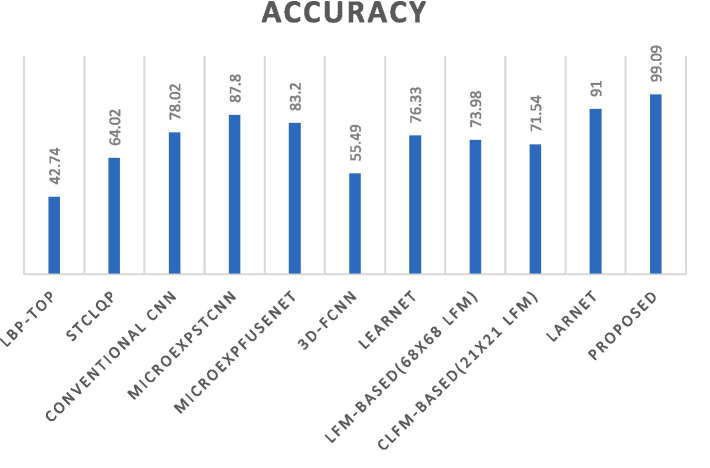
Comparison between state-of-the-art techniques with proposed method.

The facial landmarks frame-wise were extracted by a 2D landmark feature map (LFM) and CLFM (47), which were subsequently fed directly into the CNN-LSTM composite architecture. This methodology places emphasis on all facial areas, resulting in a degradation of the outcomes rather than accurately identifying the primary focal points within the film. Therefore, the outcomes were not entirely precise and exhibit limited proficiency in identifying a small range of emotions. Furthermore, The Lossless Attention Residual Network (LARnet) framework (19) was proposed which exhibits a modest improvement over the 3D model presented in the (44) study. LARNet framework described as a computational model that employs feature fusion extraction techniques on distinct facial regions to effectively identify micro expressions with high precision. The HybridMicroNet framework, as depicted in Figure 4, exhibits superior performance compared to existing techniques in terms of accuracy.

### 4.5 Challenges

The implementation and integration of HybridMicroNet architecture provide significant prospects and intricate challenges in the discipline of accurate and effective face micro-expression recognition. This section highlights and delves into multifaceted challenges encompassing implementation, model design, dataset characteristics, and result evaluation. The issues encompass a diverse array of subjects, spanning from the intricacies of optimizing and scaling models to the ethical implications of utilizing data. Furthermore, the presence of dataset restrictions, model interpretability, and assessment measures provide substantial challenges that require meticulous consideration and innovative resolutions. The enhancement of facial micro-expression recognition models can be significantly advanced through a comprehensive analysis and resolution of the challenges pertaining to their feasibility, accuracy, and practicality. The proposed HybridMicroNet architecture presents significant challenges and considerations pertaining to its implementation, model design, dataset selection, and the resulting outcomes.

1. Implementation Challenges:
  1. Data Pre- processing: The attainment of real-time performance, particularly in the realm of video analysis and emotion identification, is a notable challenge. The computing requirements associated with the processing of many frames per second, while simultaneously ensuring correctness, may impose a burden on available resources and impede the timely implementation of the system (20).
  2. Scalability and Optimization: The attainment of scalability and optimization in the proposed method, with regards to properly managing larger datasets or accommodating varied input resolutions, may provide complications in practice. Achieving scalability necessitates the utmost significance of effectively managing memory, processing resources, and model inference time (48).
2. Dataset Considerations:

The limited size of accessible datasets for micro-expression identification could restrict the model’s capacity to effectively learn a wide range of intricate and subtle expressions. In spite of concerted endeavors to promote diversity within datasets, it is possible for them to possess intrinsic biases, which might subsequently impact the model’s capacity to effectively apply to real-world situations. The problem lies in effectively addressing biases associated with race, age, gender, and emotional expressions present in the dataset (18).

1. Model Design:

The HybridMicroNet is a neural network model that combines architectural components from VGG-16 and ResNet. Achieving optimal performance with this model requires meticulous modification of hyperparameters such as learning rate, layer structure, batch size, frame size, and regularization approaches (49).

1. Cross-Dataset Evaluation:

The assessment of the model’s performance on different datasets with distinctive characteristics presents difficulties in evaluating its adaptability and robustness in various contexts. The validation of the model across different datasets is essential for gaining insights into its capacity to generalize beyond the training data (16).

## 5 Case study

**Figure 5:**
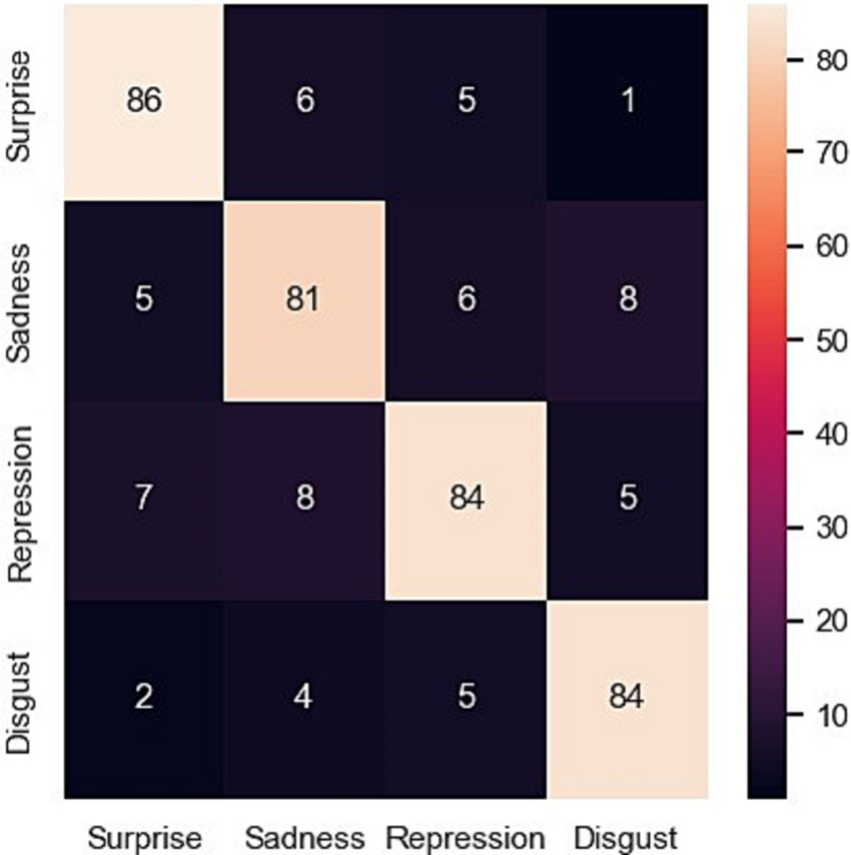
Confusion matrix for use case analysis.

Depression is a challenging mental health condition that affects millions of people all over the world. To be able to provide adequate help to individuals who are in need, early detection and intervention at the proper time are both essential. This case study investigates the use of a proposed model for emotion recognition. The experiment achieved 99.08% accuracy for CASME-II dataset and 97.62 accuracy for SAMM dataset to predict the emotions, which validated with the true label given in both datasets. Experimentation on real-time development of the trained model on the data of depressive patients was obtained from the YouTube site. This dataset comprises videos of people who had been clinically diagnosed with depression as well as some with stress, major depressive disorder, and post-traumatic stress disorder. The data was properly checked to ensure that it included a sample that was representative of all relevant demographics, including gender, age, and ethnicity. Testing on the depression patient dataset provides the results that depressed individuals express more negative emotions such as disgust, sadness, repression, and surprise, as shown in Figure 5.

The challenge of detecting and addressing depression, a prevalent mental health condition impacting millions globally, necessitates timely intervention and accurate detection methods. This case study delves into the exploration of an innovative model for emotion recognition, particularly focusing on its application in identifying emotions among individuals diagnosed with depression. However, during the evaluation of the model using real-time data obtained from individuals who have been diagnosed with mental illness and sourced from YouTube videos, many shortcomings were identified and are outlined as follows:

### 5.1 Dataset representation

Despite attempts to achieve diversity in terms of gender, age, and ethnicity, it is acknowledged that the dataset may not comprehensively encompass the wide range of variances observed among individuals with depression. The extent to which the dataset accurately represents the whole range of depressed symptoms and severity levels may be constrained, which could have implications for the model’s capacity to be applied to a broader population (18).

### 5.2 External validity

The reliance on a specific dataset sourced from YouTube raises concerns regarding the generalizability of the conclusions, since it may not sufficiently capture the broader population of individuals affected by depression. The model’s performance may demonstrate fluctuation when applied to datasets with various characteristics or when tested in real-world scenarios, hence impacting its external validity and generalizability (50).

### 5.3 Challenges in real-time applications

Although the model demonstrated a high level of accuracy in offline trials, its implementation in real-time scenarios may encounter obstacles pertaining to processing resources, latency, and the ability to adapt to dynamic, real-world conditions (18, 51).

### 5.4 Labeling accuracy

The study presents a significant level of precision in forecasting emotions; however, it is crucial to recognize the potential challenges linked to accurately classifying emotions, particularly in the context of mental health disorders like depression. The veracity of the labels that depict the actuality within the dataset could potentially be affected by the subjective nature of interpreting emotional expression and any biases in the labeling process (52).

### 5.5 Ethical considerations

The utilization of data derived from YouTube, specifically videos featuring persons who have received clinical diagnoses for mental health illnesses, gives rise to ethical considerations pertaining to issues of consent, privacy, and the safeguarding of data confidentiality (53).

## 6 Conclusion and future scope

The investigation of mental health detection using micro-expressions and machine learning has shown tremendous potential in the field of mental health evaluation and support. Micro-expressions represent brief and involuntary facial expressions that people make without even realizing it, and they can reveal important information about how people are feeling. It is possible to teach machine learning algorithms to recognize micro-expressions with a high degree of accuracy, even when these expressions are ephemeral or subtle. This case study has proved the potential of using micro-expressions as a helpful source of information for spotting indicators of many mental health issues, including depression and anxiety.

Traditional techniques of assessing mental health, such as self-report questionnaires and clinical interviews, have their fair share of flaws, some of which may be circumvented with the help of this methodology. Self-report questionnaires have a risk of producing erroneous results because respondents may be unaware of their mental health issues or may be reluctant to disclose them. Clinical interviews may prove expensive as well as time-consuming, and there is no guarantee that everyone who requires them will have access to them.

The findings obtained by implementing the HybridMicroNet architecture show that it has the potential to recognize face micro-expressions, which is an important area of research. The model demonstrates great proficiency in identifying minor emotional cues, as seen by its accuracy rates of 99.08% on the CASME-II dataset and 97.62% on the SAMM dataset, respectively. Its multi-phase technique, which involves face detection based on the Haar cascade and feature extraction using models such as VGG-16 and ResNet, is able to effectively capture subtle facial traits that are essential for micro-expression identification. The architecture is resilient in the sense that it can recognize a wide variety of feelings, which reflects its adaptability to a number of different emotional states. Understanding the functionality of the model is made easier by in-depth insights into the configuration of the model, which provide light on the architectural components of the model. Evaluation metrics, such as confusion matrices and accuracy measurements across a variety of emotions, provide a holistic perspective on the performance of the model. However, the study draws attention to obstacles such as limitations of datasets, and the model’s interpretability, highlighting the need for future development to improve accuracy and applicability in real-world circumstances.

The findings of the proposed architecture, that exhibit remarkable accuracy rates in facial micro-expressions identification, have potential implications for mental health, particularly in the post COVID-19 era. This technical development has a tremendous amount of potential applications in the field of mental health, and it offers an opportunity gateway for early identification and intervention. Tools such as the HybridMicroNet architecture could prove to be essential tools as nations struggle to recover from the effects of the COVID-19 pandemic, which has led to an increase in the prevalence of mental health problems.

## Data availability statement

The original contributions presented in the study are included in the article/supplementary material, further inquiries can be directed to the corresponding authors.

## Author contributions

DS: Conceptualization, Data curation, Investigation, Methodology, Writing – original draft, Writing – review & editing. JS: Conceptualization, Methodology, Software, Supervision, Writing – original draft, Writing – review & editing. BS: Conceptualization, Investigation, Validation, Writing – original draft, Writing – review & editing. FA: Conceptualization, Investigation, Methodology, Supervision, Writing – original draft, Writing – review & editing. AA: Conceptualization, Funding acquisition, Investigation, Methodology, Writing – original draft, Writing – review & editing. MA: Conceptualization, Data curation, Methodology, Software, Writing – original draft, Writing – review & editing.
